# Phenotyping and Genotyping Analyses Reveal the Spread of *Puccinia striiformis* f. sp. *tritici* Aeciospores From Susceptible Barberry to Wheat in Qinghai of China

**DOI:** 10.3389/fpls.2021.764304

**Published:** 2021-12-17

**Authors:** Wen Chen, ZeDong Zhang, Xinyao Ma, Gensheng Zhang, Qiang Yao, Zhensheng Kang, Jie Zhao

**Affiliations:** ^1^State Key Laboratory of Crop Stress Biology for Arid Areas, College of Plant Protection, Northwest A&F University, Yangling, China; ^2^Guizhou Academy of Agricultural Sciences, Institute of Plant Protection, Guiyang, China; ^3^Academy of Agricultural and Forestry Sciences, Qinghai University, Xining, China

**Keywords:** wheat stripe rust, *Puccinia striiformis* f. sp. *tritici*, barberry, sexual reproduction, virulence, genotype, genetic diversity, inoculum

## Abstract

*Puccinia striiformis* f. sp. *tritici* Eriks., the cause of wheat yellow or stripe rust on wheat, undergoes sexual reproduction on barberry, but it is unclear if barberry plays any role in stripe rust epidemics under natural conditions. *P. striiformis* f. sp. *tritici* was isolated from its alternate host barberry (*Berberis* spp.) and primary host wheat in the vicinity of barberry by inoculation of aeciospores and urediniospores on Mingxian 169 cultivar in Qinghai province of China in 2018. The *P. striiformis* f. sp. *tritici* isolates from barberry and wheat were characterized to virulence patterns by inoculation on 24 differentials bearing *Yr* gene under control conditions and analyzed using 12 polymorphic simple sequence repeat (SSR) markers. The occurrence frequency of *P. striiformis* f. sp. *tritici* on barberry was 1.87% by inoculation aecia, collected from barberry on Mingxian 169 of wheat. A close virulence relationship was presented between *P. striiformis* f. sp. *tritici* isolates from both barberry and wheat based on virulence simple matching coefficient and principal coordinates analysis (PCoA). Additionally, the same genetic ancestry, based on structure analysis by STRUCTURE program and genetic relationship analyses using discriminant analysis of principal components and PCoA, was shared between *P. striiformis* f. sp. *tritici* isolates from barberry and those from wheat. Together, all the results indicated that the role of barberry in providing aeciospores as an inoculum source causing wheat stripe rust epidemic in Qinghai in spring is of considerable importance.

## Introduction

*Puccinia striiformis* Westend. f. sp. *tritici* Eriks., the cause of wheat stripe (yellow) rust, is a macrocyclic rust with all five different spore stages of urediniospore, teliospore, basidiospore, pycniospore, and aeciospore in the life cycle (Jin et al., 2010; Zhao et al., 2013, 2016). The rust fungus is heteroecious and can infect barberry (*Berberis* spp., mainly) or Oregon grape (only *Mahonia aquifolium* so far) as the primary alternate host to complete its sexual cycle (Jin et al., 2010; Wang and Chen, 2013), and can infect wheat and grasses to complete its asexual cycle *via* reinfection by urediniospores (Stubbs, 1985; Hovmøller et al., 2011). So far, more than 40 barberry species and one *Mahonia* species have been reported to serve as alternate hosts for *P. striiformis* f. sp. *tritici* (Jin et al., 2010; Wang and Chen, 2013; Zhao et al., 2013; Du et al., 2019; Zhuang et al., 2019; Li et al., 2020). Some studies have demonstrated that the sexual reproduction of *P. striiformis* f. sp. *tritici* plays an important role in generating new variants and genetic diversity (Wang et al., 2012, 2018; Rodriguez-Algaba et al., 2014; Tian et al., 2016; Mehmood et al., 2020).

Although barberry infection by rusts in spring under natural conditions occurs in many parts of the world, direct evidence on the occurrence of sexual cycle of *P. striiformis* f. sp. *tritici* on wild susceptible barberry has been obtained only in China (Zhao et al., 2013), but not in other countries (Berlin et al., 2013; Wang et al., 2015; Mehmood et al., 2019). Chinese researchers have recovered many *P. striiformis* f. sp. *tritici* isolates from different naturally rusted barberry species in different provinces of China (Zhao et al., 2013; Li et al., 2016; Wang et al., 2016). Thus, susceptible barberry could release aeciospores as primary inocula or inocula to wheat to cause stripe rust in those regions. Zhao (2017) reported, based on phenotyping and genotyping analyses, that susceptible barberry bushes play an important role in the occurrence of wheat stripe rust in spring in western Shaanxi of China. However, in the U. S. Pacific Northwest, Wang et al. (2015) proved that barberry is functional for *P. graminis* f. sp. *tritici* rather than *P. striiformis* f. sp. *tritici*. Berlin et al. (2012, 2013) concluded that in Sweden barberry is important for maintaining *P. graminis* populations on rye and oats.

Qinghai is an important oversummering region for *P. striiformis* f. sp. *tritici* in China and a hot spot for the rust pathogen with high genetic diversity. A study by Du et al. (2019) reported that there are at least 10 *Berberis* species in Qinghai, and all of them can serve as alternate hosts for *P. striiformis* f. sp. *tritici*. Importantly, based on our field surveys, barberry bushes are commonly rusted in spring in this region. However, it is unknown whether *P. striiformis* f. sp. *tritici* can infect susceptible barberry to complete their sexual cycle under natural conditions, and whether barberry could release aeciospores to wheat causing stripe rust. Therefore, in this work, we aimed at determining infection of *P. striiformis* f. sp. *tritici* on barberry and verifying whether barberry plays a role in occurrence of wheat stripe rust in Qinghai based on phenotyping and genotyping.

## Materials and Methods

### Aecial Samples From Barberry and Stripe Rust-Diseased Leaf Samples From Wheat

**Figure 1:**
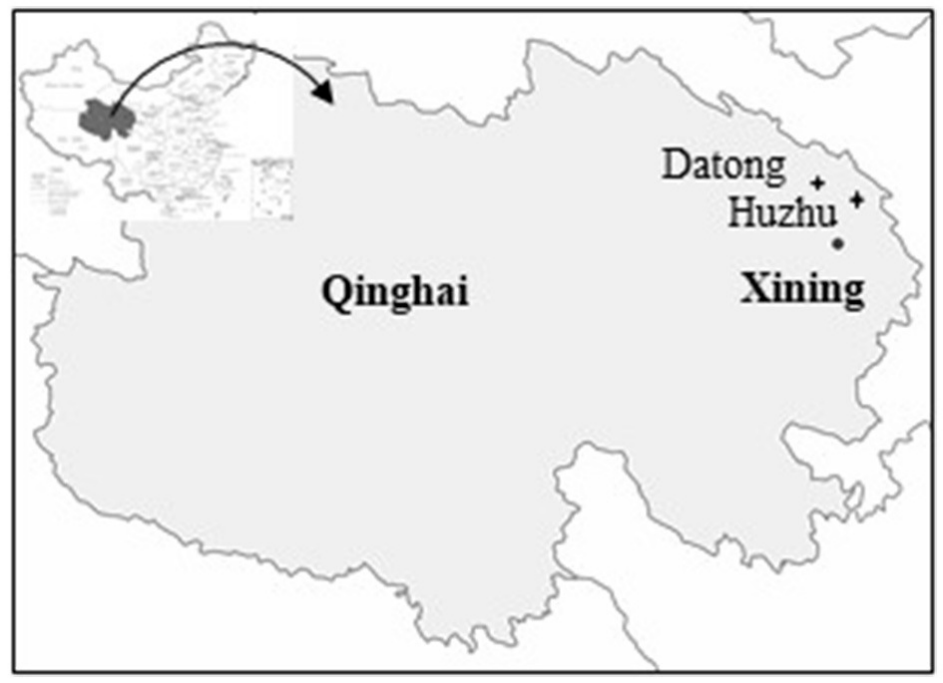
A map showing sampling location in Qinghai of China.

Aecial samples were collected from barberry (*Berberis diaphana* and *B. dubia*) bushes in Huzhu and Datong counties of Qinghai province from June 11, 2018 to June 25, 2018 (Figure 1). Sampling was performed at an interval space of ~5 m between barberry bushes as a sampling site. A short shoot with leaves bearing aecia were cut off from barberry and then put inside a sampling paper bag. To keep the freshness of aecial samples, the collected samples were brought back to the laboratory in a short time for inoculation. Leaf samples of wheat stripe rust were collected from wheat in a radius of 100 m far from the rusted barberry at 1 week after emergence of aecia on barberry tissues at the mid-to-late June of 2018. Each of the leaf samples was placed in a sampling paper bag, kept at the room temperatures for drying completely, and stored in a desiccator at low temperatures (4–5°C) until use.

### Inoculation

To obtain *P. striiformis* f. sp. *tritici* isolates from rusted barberry, individual leaf of 10-day old seedlings of wheat cultivar Mingxian 169, susceptible to Chinese races of *P. striiformis* f. sp. *tritici* identified so far, were used to inoculate with aeciospores of an individual aecial lesion. Aeciospore suspension was made by adding one drop of deionized water and mixing it well on a clean glass slide. Inoculated wheat plants were transferred to a dew chamber (E-36L2, Percival, IA, USA) and incubated in dark for 36 h at 10°C. After inoculation, wheat plants were moved into a growth chamber in a condition-controlled greenhouse and cultivated in a dual system of 16-h light at 16°C and 8-h night at 13°C.

For each of the wheat stripe rust samples, the routine operation method, described by Liu et al. (2021), was used to establish a pure isolate of single uredinium and to increase urediniospores.

### Obtainment of Pure Isolates

Uredinia produced on leaves of the wheat plants were checked 15–20 days post-inoculation of aeciospores. To obtain a pure isolate, a single pustule (uredinium), before breaking the epidermis of a wheat leaf, was picked using an office pin to be transferred to a leaf of a new wheat plant (cv. Mingxian 169). The same conditions mentioned above were used to incubate single uredinium after transferring. Fresh urediniospores were collected into a glass tube by gently tapping. Inoculation was repeated for multiplying enough urediniospores.

### Virulence Testing

The differential host set of 24 single *Yr* gene lines (SGLs), AvSYr1NIL(*Yr1*), including AvSYr5NIL(*Yr5*), AvSYr6NIL(*Yr6*), AvSYr7NIL(*Yr7*), AvSYr8NIL(*Yr8*), AvSYr9NIL(*Yr9*), AvSYr10NIL(*Yr10*), AvSYr15NIL(*Yr15*), AvSYr17NIL(*Yr17*), AvSYr27NIL(*Yr27*), AvSYr32NIL(*Yr32*), AvS/IDO377s F3-41-1 (*Yr43*), AvS/Zak 1-1-35-line1 (*Yr44*), AvSYrSPNIL (*YrSP*), AvSYrTr1NIL(*YrTr1*), AvS/Exp1/1-1Li (*YrExp2*), Tyee (*Yr76*), Kalyansona (*Yr2*), Hugenoot (*Yr25*), AvSYr28NIL (*Yr28*), AvSYr29NIL (*Yr29*), Vilmorin 23 (*Yr3*), AvSYrANIL (*YrA*), and 92R137 (*Yr26*), was used to identify avirulence and virulence patterns of isolates. A mixture of urediniospores and talc powder (v:v = 1:20) was used to inoculate seedlings (two-leaf stage) of the differentials according to the method described by Liu et al. (2021). After inoculation, the same conditions mentioned above were used. A 0–9 scale was used to assess avirulence and virulence phenotypes of isolates (Johnson et al., 1972; Line and Qayoum, 1992). Infection types of 0–6 were considered to be avirulent, and those of 7–9 as virulent. Virulence patterns, virulence frequency, and Kosman diversity index (Kosman and Leonard, 2007) of isolates were determined using the software VAT 1.0 (Schachtel et al., 2012).

### Microsatellite Analysis

Cetyltrimethylammonium bromide (CTAB) method with modification was used to extract genomic DNA of urediniospores of isolates (Aljanabi and Martinez, 1997). Concentration of DNA solution was diluted to 50 ng/ml for polymerase chain reaction (PCR) amplification with simple sequence repeats (SSR) markers. Totally, 12 pairs of SSR primers, CPS11, CPS32, and CPS34 (Chen et al., 2009), RJ3N and RJ11N (Bahri et al., 2009), RJ27 (Enjalbert et al., 2002), *Pst*P031(Cheng et al., 2012), SUNI*Pst*15-30, SUNI*Pst*11-10, SUNI*Pst*11-44, and SUNI*Pst*10-48 (Tian et al., 2016), and WU6 (Ali et al., 2011), were used for the genotyping of isolates. Primers were synthesized by Sangon Biotech (Shanghai) Co., Ltd. (Shanghai, China), and the 5′-end of forward primers were fluorescently labeled. PCR products were analyzed on a DNA Analyzer (3730XL, Applied Biosystems, Waltham, MA, USA). SSR amplicons were evaluated using the software GeneMarker HID (Holland and Parson, 2011). Multilocus genotypes (MLGs) were detected based on microsatellite loci using GenClone 2.0 program (Arnaud-haond and Belkhir, 2007). The expected heterozygosity, Shannon's diversity index, and allele analysis in populations were conducted using GenAlEx 6.5 (Peakall and Smouse, 2012), and analysis of molecular variance (AMOVA) was used to evaluate microsatellite variation. Using NTSYS 2.10e program, a similarity matrix was generated based on the simple matching (SM) coefficient in the Qualitative module. The sequential, agglomerative, hierarchical, and nested (SAHN) clustering method was chosen for constructing a dendrogram for isolates by the unweighted pair group method with the arithmetic means (UPGMA) (Rohl, 2000).

Patterns of population structure and admixture were analyzed using the Bayesian model method, and implemented in software STRUCTURE 2.2 (Pritchard et al., 2000). The Monte Carlo Markov Chain scheme was run, as recommended by 100,000 and *K* value ranging from 1 to 10 with at least 100 repetitions to check the convergence of likelihood value for each *K* value. The optimal number of population subdivisions was determined by plotting the graph of estimated values of logarithm likelihood for each *K* value, and the maximum logarithm likelihood was used. For each *K* value, the Large *K* Greedy algorithm implemented in the program CLUMPP version 1.1 was used to search for the best alignment of multiple replicate cluster analyses (Jakobsson and Rosenberg, 2007). The population structure was visualized using DISTRUCT version 1.1 (Rosenberg, 2004). To supplement the output from STRUCTURE, discriminant analysis of principal components (DAPC) was performed in the adegenet package in R environment (Jombart et al., 2010).

## Results

### Inoculation of Single Aecium Collection From Barberry

**Figure 2:**
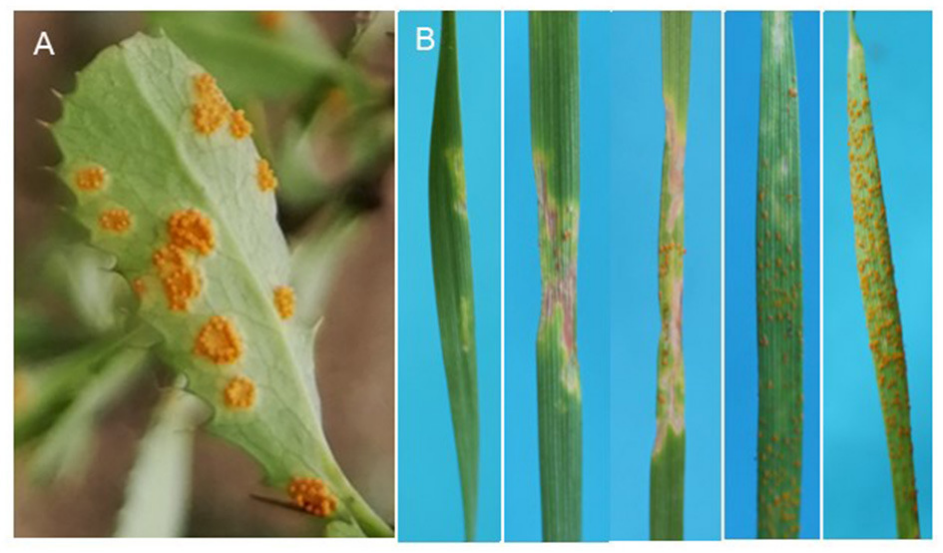
Different infection types on leaves of wheat cv. Mingxian 169 after inoculation with aeciospores from aecial samples collected from naturally rusted barberry. **(A)** A leaf sample showing aecia produced on barberry (*Berberis diaphana*). **(B)** Infection types on leaves of wheat cv. Mingxian 169 after inoculation with aeciospores of aecial samples.

Pycnia was initially observed on barberry tissues in the early June, and aecia was produced from middle June to early August in Qinghai (Figure 2A). Totally, 375 single aecium, collected from 52 rust-infected barberry bushes at 11 sampling sites located in Huzhu and Datong counties of Qinghai, were inoculated on wheat cv. Mingxian 169. Seven (1.87%) of all the 375 collected aecial samples produced typical *P. striiformis* f. sp. *tritici* uredinial symptoms on wheat leaves with different infection types (Figure 2B). As a result, a population of 29 single-uredinium isolates, derived from the seven aecial samples, were established for subsequent analysis. Meanwhile, 54 pure isolates were obtained from sample collections of wheat plants adjacent to barberry bushes.

### Virulence Characterization and Genetic Variation

**Figure 3:**
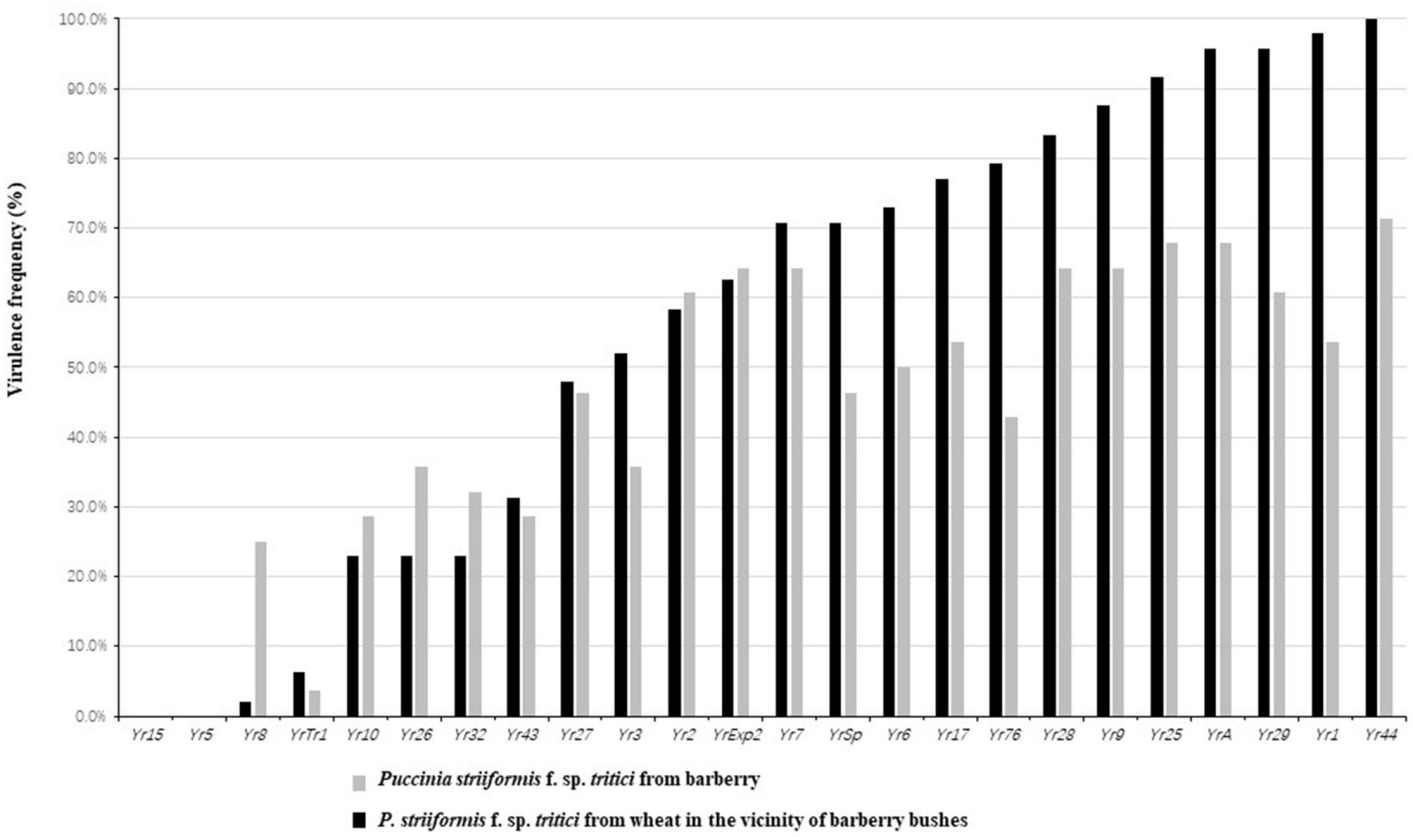
Virulence frequency of *Puccinia striiformis* f. sp. *tritici* isolates obtained from barberry bushes and wheat plants adjacent to barberry on the 24 *Yr* single gene lines.

Phenotype testing indicated that frequency of all isolates from barberry virulent to *Yr*. genes, except for *Yr44*, were <70%. Conversely, isolates from barberry showed higher virulence frequency than those from wheat at several resistance loci, including *Yr8, Yr10, Yr26, Yr32, Yr2*, and *YrExp2*. Particularly, frequency of isolates from barberry virulent to *Yr8* was remarkably higher than that from wheat plants close to barberry. Comparatively, most of the isolates (>70%) from wheat were virulent to *Yr7, YrSP, Yr6, Yr17, Yr76* (*YrTye*)*, Yr28, Yr9, Yr25, YrA, Yr1, Yr29, Yr1*, and *Yr44*. Regardless of isolates from barberry or from wheat, all of them were avirulent to *Yr5* and *Yr15* (Figure 3).

Both *P. striiformis* f. sp. *triatic* populations from barberry and wheat showed a high level of diversity in virulence. In total, 28 out of 29 (one isolate invalid) isolates from barberry produced 17 virulence patterns (Table 1), and 8 isolates of them were avirulent to all of the 24 SGLs (Supplementary Table 1). In contrast, 44 virulence patterns were detected in a population of 48 out of 54, of which 6 were invalid isolates from wheat, significantly higher than those from barberry (Table 1; Supplementary Table 1). The population from barberry, with *Nei*'s diversity index (*Hs* = 0.402) and Kosman index (*K* = 0.649), showed a higher virulence diversity than that from wheat with a *Hs* value of 0.265 and a *K* value of 0.384 (Table 1). Genotyping analysis showed that 24 MLGs were identified among 29 isolates from barberry, and that 38 were detectable among 54 isolates from wheat (Table 1). Both populations showed high diverse MLGs, and MLG ratio of the former (82.8%) was higher than those of the latter (70.4%). However, no same MLGs were detected in two populations from barberry and wheat. Based on genotypic diversity analysis, the mean Shannon's diversity index (*I*) and expected heterozygosity (*He*) of the population from barberry was 0.691 and 0.362, and those from wheat were 0.635 and 0.343, respectively (Table 1). The number of the virulence patterns and MLGs, and virulence and genetic diversity index of two populations from barberry and wheat were obviously high, indicating that the two populations were not strictly clonal.

**Table 1:** Population genetic data of *Puccinia striiformis* f. sp. *tritici* isolates from barberry bushes and wheat plants in Nanmengxia, Huzhu, Qinghai.

**Plant**	**Sampling site (longitude and latitude)**	**Sampling date**	**No.^a^**	**Phenotype**			**No.^b^**	**Genotype**			
				**VP^c^**	***Hs*** **^d^**	***K*** **^e^**		**No. of MLGs^f^**	***CF*** **^g^**	***I*** **^h^**	***He*** **^i^**
Barberry	Nanmengxia town, Huzhu county (101.896472, 36.959524)	June-11-2018	16	8	0.347	0.557	16	16	0	0.698	0.380
		June-25-2018	12	9	0.256	0.389	13	10	0.231	0.486	0.280
		Total	28	17	0.402	0.649	29	24	0.172	0.691	0.362
Wheat	Nanmengxia town, Huzhu county (101.896472, 36.959524)	June-17-2018	7	7	0.255	0.381	7	7	0	0.530	0.315
		June-25-2018	41	39	0.264	0.382	47	35	0.255	0.632	0.344
		Total	48	44	0.265	0.384	54	38	0.296	0.635	0.343

a *The number of successfully virulence identification samples*.

b *The number of samples genotyped successfully*.

c *VP, the number of virulence patterns*.

d *Hs, Nei divrsity*.

e *K, Kosman index*.

f *MLGs, multilocus genotypes*.

g *CF, 1-[(number of MLGs)/(total number of isolates)]*.

h *I, Shannon's Information Index*.

i *He, expected heterozygosity*.

**Figure 4:**
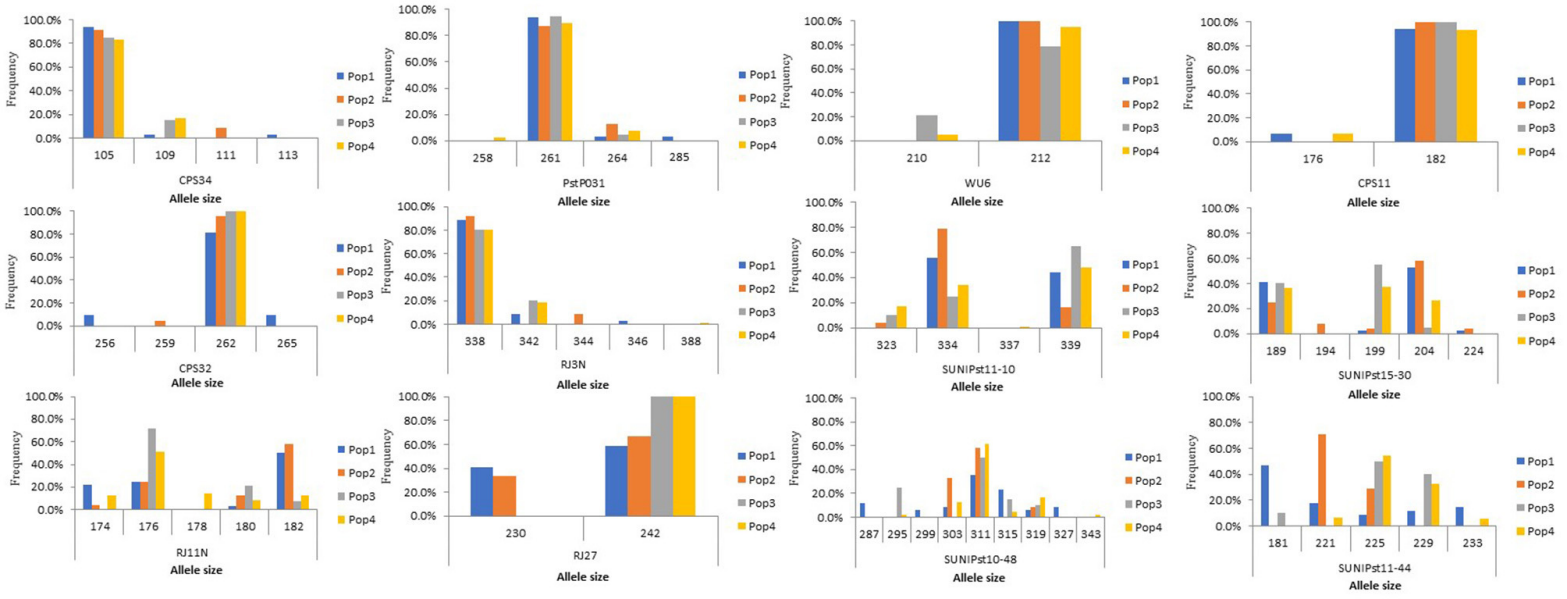
Allele frequency for markers of CPS34, *Pst*P031, WU6, CPS11, CPS32, RJ3N, SUNI*Pst*11-10, SUNI*Pst*15-30, RJ11N, RJ27, SUNI*Pst*10-48, SUNI*Pst*11-44 in *Puccinia striiformis* f. sp. *tritici* populations from barberry bushes (Pop1-Pop2) and wheat plants close to the barberry bushes (Pop3-Pop4).

Detection of the allelic variation uncovered that thirty same alleles were observed among 12 loci between isolates from barberry and those from wheat. The allele frequencies of isolates from barberry and wheat are presented in Figure 4. Although isolates from barberry and wheat shared most of the alleles, unique alleles at 7 SSR loci were detected among the isolates from barberry. These unique alleles included 111 and 113 at locus CPS34; 285 at locus *Pst*P031; 256, 259, and 265 at locus CPS32; 344 and 346 at locus RJ3N; 194 and 224 at locus SUNI*Pst*15-30; 230 at locus RJ27; 287, 299, and 327 at locus SUNI*Pst*10-48. Similarly, some alleles, including 258 at locus *Pst*P031, 210 at locus WU6, 337 at locus SUNI*Pst*11-10, and 178 at locus RJ11N, were unique in the population from wheat, but not in that from barberry. For each locus, the number of alleles of the population from barberry was higher than that from wheat. All results mentioned above indicated that alleles of population from barberry were more abundant than that from wheat, and overlapping alleles were detectable between the population from wheat and those from barberry.

To examine the variation between the populations from barberry and wheat (Table 2), the AMOVA was used, and it showed that the variation between the populations from different hosts was 11.78%, that among populations from a common host was 4.17%, and that within populations was 84.05%, respectively. These results indicated that there was not a significant differentiation between the populations from barberry host and wheat host, and a large proportion of the variation presented within the population.

**Table 2:** Analysis of molecular variance (AMOVA) among and within the *Puccinia striiformis* f. sp. *tritici* populations from barberry bushes and wheat plants based on the simple sequence repeat markers.

**Source**	**AMOVA parameters^a^**					
	***DF***	***SS***	***MS***	***EV***	***PV*** **(%)**	***P***
Between hosts	1	28.90	28.90	0.29	11.78	<0.001
Among populations	2	10.46	5.23	0.10.	4.17	<0.001
Within population	162	337.98	2.036	2.09	84.05	<0.001
Total	165	377.34	–	2.48	100.00	

a *DF, degree of freedom; SS, sum of squared deviation; EV, estimated variance; PV, percentage of variance; P, probability*.

### Relationship Between Isolates From Barberry and Wheat Based on Phenotyping and Genotyping Analyses

**Figure 5:**
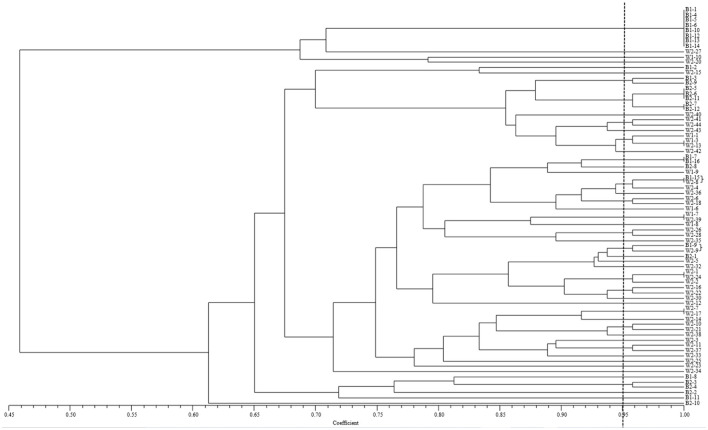
Clustering based on the simple matching (SM) coefficient of *Puccinia striiformis* f. sp. *tritici* isolates virulent to the 24 single *Yr* gene lines as differentials. Isolates of B1-1 to B1-16 of *Puccinia striiformis* f. sp. *tritici* recovered from rusted barberry bushes on 11 June, 2018, and those of B2-1 to B2-13 from barberry on 25 June, 2018. Isolates of W1-1 to W1-10 of *P. striiformis* f. sp. *tritici* collected from wheat in the vicinity of barberry bushes on 17 June, 2018, and those of W2-1 to W2-44 from nearby wheat on 25 June, 2018.

**Figure 6:**
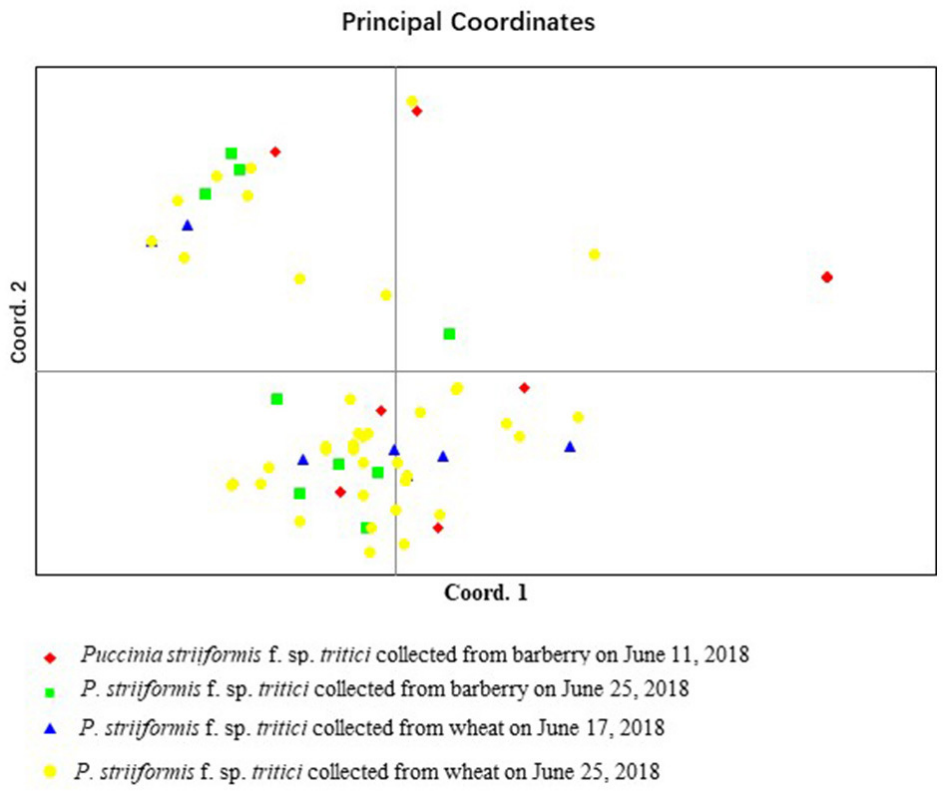
Principal coordinates analysis (PCoA) based on the calculated virulence differences between each of *Puccinia striiformis* f. sp. *tritici* isolates. The first axis represents 43.22% and the second represents 17.44% of the total virulence differences between the individual isolates.

Phenotyping analysis, based on virulence in the NTSYS 2.10e program, indicated that isolate B1-15 from barberry and isolate W2-8 from wheat had the same virulence pattern, and that isolate B1-9 from barberry and isolate W2-9 from wheat were clustered in a clade with the simple matching coefficient value of 0.95 (Figure 5). In terms of the calculated virulence differences between each of the isolates, principal coordinates analysis (PCoA) with GenAlEx 6.5 software indicated that isolates from barberry were clustered together with those from wheat, and that 43.22 and 17.44% of the total virulence differences between the individuals of all isolates are represented in the first axis and the second axis, respectively (Figure 6). All results indicated that a close relationship existed between the populations from barberry and wheat in virulence.

**Figure 7:**
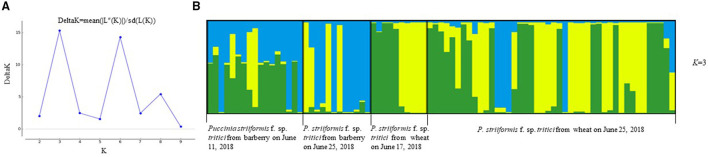
Clustering of 83 *P. striiformis* f. sp. *tritici* isolates from barberry bushes and wheat plants in field to genotypic groups for the optimal *K*-value (*K* = 3). **(A)** Values of logarithm likelihood based on analysis by structure program. **(B)** Each of color bars represents a kind of genotypic group.

**Figure 8:**
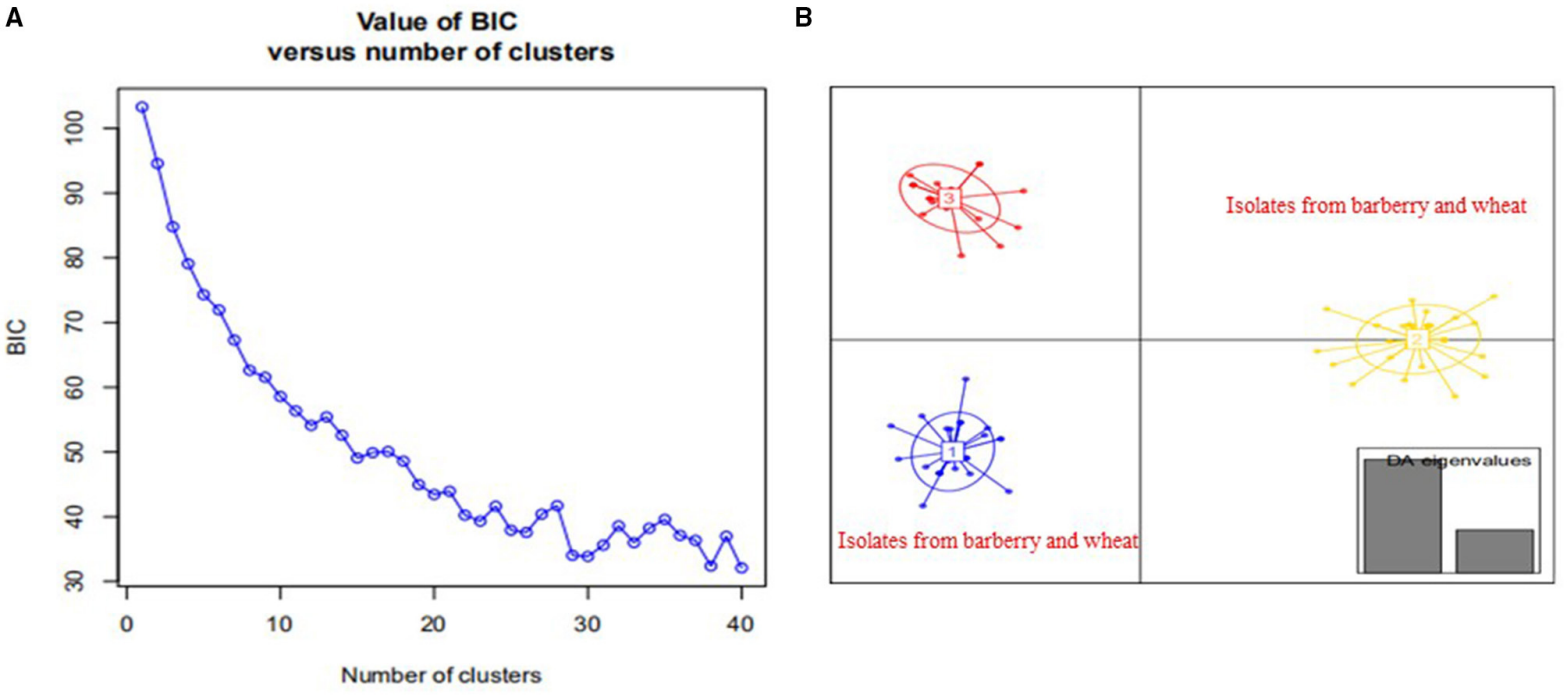
Discriminant analysis of principal components (DAPC) analysis of *Puccinia striiformis* f. sp. *tritici* populations sampled from barberry bushes and nearby wheat plants. **(A)** Value of Bayesian information criteria vs. the number of clusters for all *P. striiformis* f. sp. *tritici* isolates. **(B)** Scatter-plot of the *P. striiformis* f. sp. *tritici* isolates from barberry and wheat plants, and eigenvalues of the analysis are displayed in gray bars in the right corner.

Population structure analysis with 12 pairs of polymorphic SSR primers revealed a close relationship between wheat population and barberry population. Using the structure program, the number of optimal inferred cluster (*K*) was 3 (Figure 7A). When *K* = 3, isolates from barberry showed that ancestry primarily derived from two inferred clusters (blue and green bars), either single or combined, and that isolates from wheat showed ancestry mainly originated from two clusters (green and yellow bars). Some isolates from wheat shared identical ancestry with those from barberry (blue and yellow bars) (Figure 7B). Non-parametric DAPC for supplementary analysis using structure program showed that genotypes of *P. striiformis* f. sp. *tritici* from different hosts were obviously clustered together. Based on the Bayesian information criteria in the DAPC analyses, the whole population generated 3 clusters (Figure 8A). Isolates from barberry and those from wheat were grouped into one subcluster in group 1 or group 2 (Figure 8B). This indicated that both *P. striiformis* f. sp. *tritici* populations from barberry and wheat in the vicinity of barberry bushes had a close genetic relationship.

**Figure 9:**
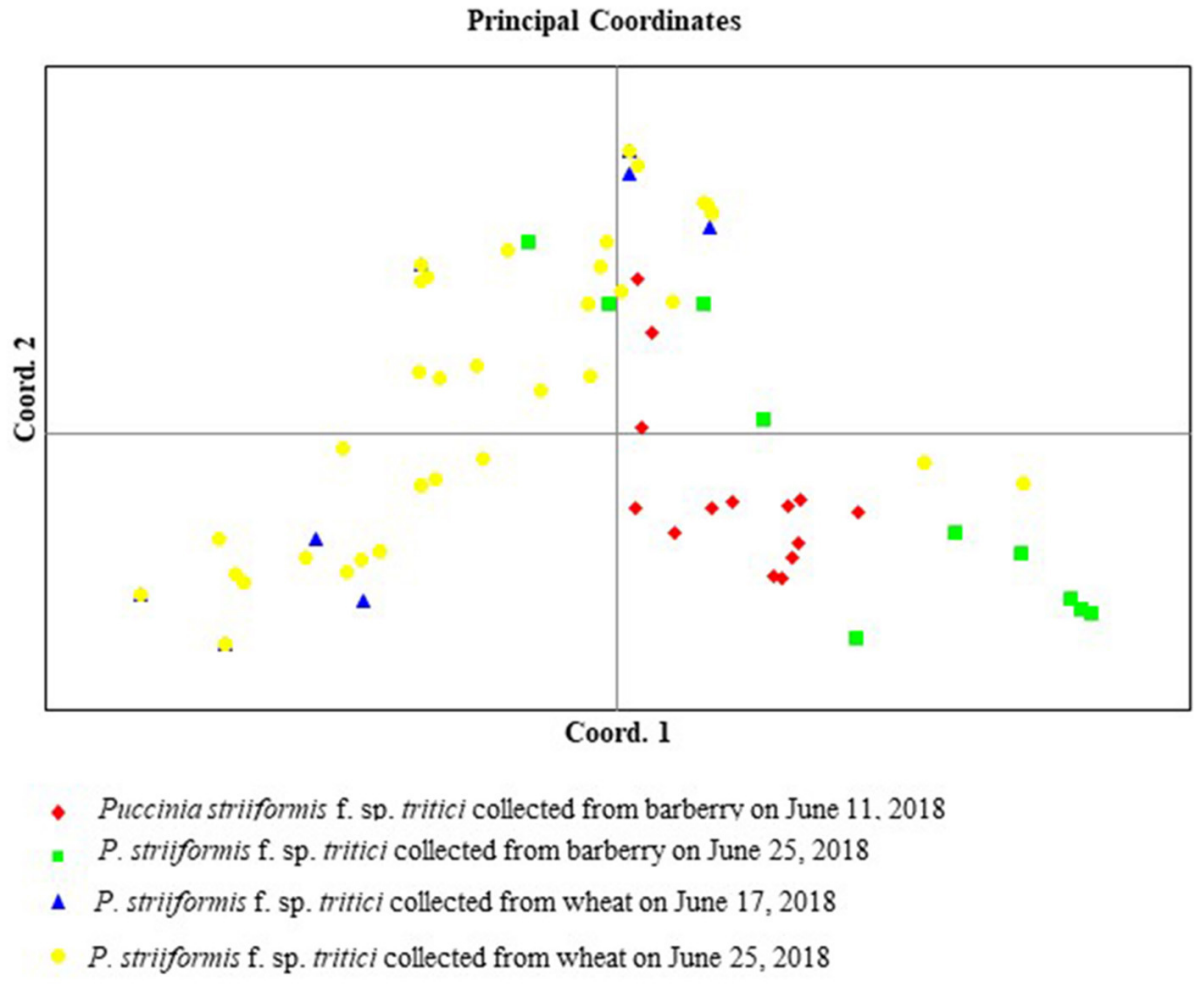
Principal coordinates analysis (PCoA) based on the calculated genetic differences between each of *Puccinia striiformis* f. sp. *tritici* isolates. The first axis represents 27.63% and the second represents 23.92% of the total genetic differences between the individual isolates.

Principal coordinates analysis showed that isolates from barberry and wheat were clustered in a large group, and that there was no obvious separate cluster for isolates from both hosts (Figure 9). The first axis of the PCoA explained 27.63% of the differences between the individual isolates, and the second explained 23.92% of the differences between the individual isolates.

## Discussion

Phenotypic and genotypic analyses revealed that single uredinium isolates populations from barberry and wheat had a large number of virulence patterns and MLGs. A higher Kosman index and expected heterozygosity indicated that virulence and genetic diversity of *P. striiformis* f. sp. *tritici* isolates from barberry and wheat was abundant. Although unique alleles in 7 loci were detected in the population from barberry, *P. striiformis* f. sp. *tritici* of single uredinium isolates from barberry appeared to possess some alleles that were identical with those from wheat. All results indicated that the stripe rust population from barberry and that from wheat may be affected mutually. Due to the lack of same MLGs and the presence of less of the same virulence patterns, a differentiation between both populations was observed.

Under natural conditions, occurrence of sexual cycle of *P. striiformis* f. sp. *tritici* on wild susceptible barberry in China is regular. To date, *P. striiformis* f. sp. *tritici* isolates have been successfully recovered from different barberry species, including *B. aggregata, B*. *polyantha, B*. *shensiana, B. soulieana, B. potaninii*, and *B*. *brachypoda*, in Gansu, Shaanxi, and Tibet in spring in four independent years (Zhao et al., 2013; Li et al., 2016; Wang et al., 2016; Zhao, 2017). Our results showed that sexual cycle of the wheat stripe rust pathogen occurs on barberry in Qinghai.

Barberry are common alternate hosts for many species of *Puccinia*. So far, more than 30 *Puccinia* species infecting barberry have been known, such as *P*. *graminis* (De Bary, 1866), *P*. *pseudostriiformis* (syn. *P. striiformis* f. *sp. poae*) (Jin et al., 2010), *P. arrhenatheri* (Naef et al., 2002), *P. achnatheri-sibirici* (Ma et al., 2020), *P. brachypodii* (Payak, 2010), *P. pygmaea, P. montanensis, P. brachypodii-phoenicoidis* (Cummins and Greene, 1966), and *P*. *striiformis* formae speciales, including f. sp. *hordei* on barley, f. sp. *agropyri* on *Agropyron*, and f. sp. *elymi* on *Elymus* (Huang et al., 2019). In this work, after inoculation, only a few of all the aecial samples infected wheat cv. Mingxian 169, with high infection types, to produce urediniospores. However, most of aecial samples produced low infection types or merely flecks without urediniospores on wheat leaves, indicating that they were other rust fungi.

Sexual reproduction is responsible for generating new variants and high genetic diversity of *P. striiformis* f. sp. *tritici*. Since the finding of the rust pathogen in 2010 (Jin et al., 2010), so far, in China more than 100 isolates of *P. striiformis* f. sp. *tritici* have been recovered from barberry by Zhao et al. (2013), Li et al. (2016), Wang et al. (2016), and Zhao (2017), most of which were new races. Genetic research by Wang et al. (2012), Tian et al. (2016), Wang et al. (2018), Du et al. (2019), and Mehmood et al. (2020) demonstrated that sexual reproduction of *P. striiformis* f. sp. *tritici* can result in arising of the new variant progeny, which differs from the parent isolates in pathogenicity. This means that sexual production is an important origin of the new variants of the wheat stripe rust pathogen. In this work, we demonstrated that in Qinghai *P. striiformis* f. sp. *tritici* infects barberry to complete sexual cycle, and barberry spread aeciospores as inocula to wheat to trigger stripe rust on the crop. Therefore, our results in this study suggest that treatment of barberry bushes, especially around wheat fields, should be involved in integrated management of wheat stripe rust in Qinghai.

## Data Availability Statement

The original contributions presented in the study are included in the article/Supplementary Material, further inquiries can be directed to the corresponding author/s.

## Author Contributions

WC was responsible for drafting the manuscript, as well as the acquisition, analysis, and interpretation of data. ZZ performed the virulence identification of the stripe rust collections. XM and GZ contributed for isolation of the rust samples from plants. QY carried out barberry investigations in fields. ZK and JZ designed the overall research and contributed to reviewing manuscript. All authors read and approved the final manuscript.

## Funding

This study was supported by the National Key Research and Development Program of China (2018YFD0200500, 2018YFD0200400), National Natural Science Foundation of China (31871918, 31960524, 32072358), National 111 Plan (No. BP0719026), the Fundamental Research Funds for the Central Universities (2452019046), and the Natural Science Basic Research Plan in Shaanxi Province of China (2020JZ-15).

## Conflict of Interest

The authors declare that the research was conducted in the absence of any commercial or financial relationships that could be construed as a potential conflict of interest.

## Publisher's Note

All claims expressed in this article are solely those of the authors and do not necessarily represent those of their affiliated organizations, or those of the publisher, the editors and the reviewers. Any product that may be evaluated in this article, or claim that may be made by its manufacturer, is not guaranteed or endorsed by the publisher.
