# Supplementary material for: Phenotyping and Genotyping Analyses Reveal the Spread of Puccinia striiformis f. sp. tritici Aeciospores From Susceptible Barberry to Wheat in Qinghai of China
Source: Front Plant Sci. 2021 Dec 17;12:764304. doi: 10.3389/fpls.2021.764304 (PMC8719489; doi:10.3389/fpls.2021.764304)
Supplement: Supplementary file 1 [file Table_1.docx]

**Supplementary Table S1.** Avirulence (A) and virulence (V) patterns of 76 out of 83 single-uredinium isolates of *Puccinia striiformis* f. sp. *tritici* from barberry bushes and wheat plants in Nanmenxia, Huzhu, Qinghai in spring in 2018

| **Isolates^a^** | **Host** | **Phenotype of isolates on single *Yr* gene lines** | | | | | | | | | | | | | | | | | | | | | | | |
| --- | --- | --- | --- | --- | --- | --- | --- | --- | --- | --- | --- | --- | --- | --- | --- | --- | --- | --- | --- | --- | --- | --- | --- | --- | --- |
|  |  | ***Yr1*** | ***Yr2*** | ***Yr3*** | ***Yr5*** | ***Yr6*** | ***Yr7*** | ***Yr8*** | ***Yr9*** | ***Yr10*** | ***Yr15*** | ***Yr17*** | ***Yr27*** | ***Yr32*** | ***Yr43*** | ***Yr44*** | ***YrSp*** | ***YrTr1*** | ***YrExp2*** | ***Yr76*** | ***Yr25*** | ***Yr28*** | ***Yr29*** | ***YrA*** | ***Yr26*** |
| B1-1 | Barberry | A | A | A | A | A | A | A | A | A | A | A | A | A | A | A | A | A | A | A | A | A | A | A | A |
| B1-2 | Barberry | A | A | A | A | A | V | A | V | V | A | A | V | V | A | V | V | A | V | A | A | V | V | V | V |
| B1-3 | Barberry | V | V | V | A | V | V | A | V | V | A | V | A | V | A | V | V | A | V | A | V | V | A | V | V |
| B1-4 | Barberry | A | A | A | A | A | A | A | A | A | A | A | A | A | A | A | A | A | A | A | A | A | A | A | A |
| B1-5 | Barberry | A | A | A | A | A | A | A | A | A | A | A | A | A | A | A | A | A | A | A | A | A | A | A | A |
| B1-6 | Barberry | A | A | A | A | A | A | A | A | A | A | A | A | A | A | A | A | A | A | A | A | A | A | A | A |
| B1-7 | Barberry | V | V | A | A | V | V | A | V | A | A | V | V | A | V | V | A | A | V | A | V | V | V | V | A |
| B1-8 | Barberry | A | V | A | A | V | V | V | A | A | A | A | A | A | V | V | A | A | V | A | V | A | V | V | A |
| B1-9 | Barberry | V | A | A | A | V | A | A | V | A | A | V | V | A | A | V | V | A | A | A | V | V | V | V | A |
| B1-10 | Barberry | A | A | A | A | A | A | A | A | A | A | A | A | A | A | A | A | A | A | A | A | A | A | A | A |
| B1-11 | Barberry | V | V | A | A | V | V | V | V | A | A | V | V | A | A | V | A | A | V | A | V | A | V | V | V |
| B1-12 | Barberry | A | A | A | A | A | A | A | A | A | A | A | A | A | A | A | A | A | A | A | A | A | A | A | A |
| B1-13 | Barberry | A | A | A | A | A | A | A | A | A | A | A | A | A | A | A | A | A | A | A | A | A | A | A | A |
| B1-14 | Barberry | A | A | A | A | A | A | A | A | A | A | A | A | A | A | A | A | A | A | A | A | A | A | A | A |
| B1-15 | Barberry | V | V | V | A | V | V | A | V | A | A | V | V | A | V | V | V | A | V | V | V | V | V | V | A |
| B1-16 | Barberry | V | V | A | A | V | V | A | V | A | A | V | V | A | V | V | A | A | V | A | V | V | V | V | A |
| B2-1 | Barberry | V | A | A | A | V | V | A | V | A | A | V | V | A | A | V | V | A | A | V | V | V | V | V | A |
| B2-2 | Barberry | A | V | V | A | V | V | V | V | A | A | V | V | V | V | V | A | V | V | V | V | V | V | V | A |
| B2-3 | Barberry | A | V | A | A | V | V | V | V | A | A | A | V | A | V | V | A | A | V | V | V | V | V | V | A |
| B2-4 | Barberry | A | V | A | A | V | V | V | V | A | A | A | V | A | V | V | V | A | V | V | V | V | V | V | A |
| B2-5 | Barberry | V | V | V | A | A | V | A | V | V | A | V | A | V | A | V | V | A | V | V | V | V | V | V | V |
| B2-6 | Barberry | V | V | V | A | A | V | A | V | V | A | V | A | V | A | V | V | A | V | V | V | V | V | V | V |
| B2-7 | Barberry | V | V | V | A | A | V | A | V | V | A | V | V | V | A | V | V | A | V | V | V | V | V | V | V |
| B2-8 | Barberry | V | V | A | A | V | V | V | V | A | A | V | V | A | V | V | V | A | V | A | V | V | V | V | A |
| B2-9 | Barberry | V | V | V | A | V | V | A | V | V | A | V | A | V | A | V | V | A | V | V | V | V | A | V | V |
| B2-10 | Barberry | V | V | V | A | V | A | V | A | A | A | A | A | A | A | V | A | A | V | V | V | V | A | V | V |
| B2-11 | Barberry | V | V | V | A | A | V | A | V | V | A | V | A | V | A | V | V | A | V | V | V | V | V | V | V |
| B2-12 | Barberry | V | V | V | A | A | V | A | V | V | A | V | V | V | A | V | V | A | V | V | V | V | V | V | V |
| B2-13 | Barberry | / ^b^ | / | / | / | / | / | / | / | / | / | / | / | / | / | / | / | / | / | / | / | / | / | / | / |
| W1-1 | Wheat | V | V | V | A | V | V | A | V | V | A | V | V | V | V | V | V | A | V | A | V | V | V | V | V |
| W1-2 | Wheat | / | / | / | / | / | / | / | / | / | / | / | / | / | / | / | / | / | / | / | / | / | / | / | / |
| W1-3 | Wheat | V | V | V | A | V | V | A | V | V | A | V | V | V | V | V | V | A | V | V | V | V | V | V | V |
| W1-4 | Wheat | / | / | / | / | / | / | / | / | / | / | / | / | / | / | / | / | / | / | / | / | / | / | / | / |
| W1-5 | Wheat | / | / | / | / | / | / | / | / | / | / | / | / | / | / | / | / | / | / | / | / | / | / | / | / |
| W1-6 | Wheat | V | A | V | A | V | V | A | V | A | A | V | V | A | V | V | V | A | V | A | V | V | V | V | A |
| W1-7 | Wheat | V | V | V | A | V | V | A | A | A | A | V | A | A | A | V | A | A | A | V | V | V | V | V | A |
| W1-8 | Wheat | V | V | V | A | V | V | A | V | A | A | V | A | A | A | V | A | A | A | A | V | A | V | V | A |
| W1-9 | Wheat | V | V | A | A | V | V | A | V | A | A | V | A | A | A | V | A | A | V | A | V | V | V | V | A |
| W1-10 | Wheat | V | V | V | A | V | A | A | A | A | A | A | V | A | A | V | A | A | A | A | V | A | A | V | A |
| W2-1 | Wheat | V | V | V | A | V | A | A | V | A | A | V | A | A | A | V | V | A | A | V | V | A | V | V | A |
| W2-2 | Wheat | V | V | V | A | V | A | A | V | A | A | V | A | A | A | V | V | A | A | V | V | V | V | V | A |
| W2-3 | Wheat | V | V | A | A | V | V | A | V | A | A | A | A | A | A | V | V | A | V | V | V | V | V | V | A |
| W2-4 | Wheat | V | V | V | A | V | V | A | V | A | A | V | V | A | V | V | V | A | V | A | V | V | V | V | A |
| W2-5 | Wheat | V | A | V | A | V | A | A | V | A | A | V | V | A | A | V | V | A | A | V | V | V | V | V | A |
| W2-6 | Wheat | V | V | V | A | V | V | A | V | A | A | V | V | A | A | V | V | A | A | V | V | V | V | V | A |
| W2-7 | Wheat | V | A | A | A | A | V | A | V | A | A | V | V | A | A | V | V | A | V | V | V | V | V | V | A |
| W2-8 | Wheat | V | V | V | A | V | V | A | V | A | A | V | V | A | V | V | V | A | V | V | V | V | V | V | A |
| W2-9 | Wheat | V | A | A | A | V | A | A | V | A | A | V | V | A | A | V | V | A | A | V | V | V | V | V | A |
| W2-10 | Wheat | V | A | A | A | A | V | A | V | A | A | V | A | A | A | V | A | A | V | V | V | V | V | V | A |
| W2-11 | Wheat | V | A | A | A | V | V | A | V | A | A | V | A | A | V | V | V | A | V | V | V | V | V | V | A |
| W2-12 | Wheat | V | A | V | A | V | A | A | A | A | A | A | A | A | A | V | V | A | A | V | V | A | V | V | A |
| W2-13 | Wheat | V | V | V | A | V | V | A | V | V | A | V | V | V | V | V | V | A | V | V | V | V | V | V | V |
| W2-14 | Wheat | V | A | A | A | A | V | A | V | V | A | V | V | V | A | V | V | A | V | V | V | V | V | V | A |
| W2-15 | Wheat | V | A | A | A | A | V | A | V | V | A | V | A | V | A | V | V | A | V | A | A | A | V | V | V |
| W2-16 | Wheat | V | V | A | A | V | A | A | V | A | A | V | V | A | A | V | V | A | A | V | V | V | V | V | A |
| W2-17 | Wheat | V | A | A | A | A | V | A | V | A | A | V | V | A | A | V | V | A | V | V | V | V | V | V | A |
| W2-18 | Wheat | V | V | V | A | V | V | A | V | A | A | V | V | A | A | V | V | A | V | V | V | V | V | V | A |
| W2-19 | Wheat | / | / | / | / | / | / | / | / | / | / | / | / | / | / | / | / | / | / | / | / | / | / | / | / |
| W2-20 | Wheat | V | V | A | A | A | A | A | V | A | A | A | A | A | A | V | A | A | A | A | V | A | V | V | A |
| W2-21 | Wheat | V | A | A | A | A | V | A | V | A | A | A | A | A | A | V | A | A | V | V | V | V | V | V | A |
| W2-22 | Wheat | V | V | A | A | V | A | A | V | A | A | V | A | A | A | V | V | A | A | V | V | V | V | V | A |
| W2-23 | Wheat | V | A | A | A | V | V | A | V | A | A | A | V | A | V | V | A | A | V | V | V | V | V | V | V |
| W2-24 | Wheat | V | V | V | A | V | A | A | V | A | A | V | A | A | A | V | V | A | A | V | V | A | V | V | A |
| W2-25 | Wheat | V | A | A | A | A | V | A | V | V | A | V | A | A | A | V | V | V | V | V | A | V | V | V | A |
| W2-26 | Wheat | V | V | V | A | A | V | A | V | A | A | V | V | A | A | V | A | A | V | V | V | V | V | V | A |
| W2-27 | Wheat | A | A | A | A | V | V | A | A | A | A | A | A | V | A | V | A | A | V | A | V | A | A | V | A |
| W2-28 | Wheat | V | V | V | A | A | A | A | V | A | A | V | V | A | A | V | A | A | V | V | V | V | V | V | A |
| W2-29 | Wheat | / | / | / | / | / | / | / | / | / | / | / | / | / | / | / | / | / | / | / | / | / | / | / | / |
| W2-30 | Wheat | V | V | A | A | V | A | A | V | A | A | V | A | A | A | V | V | A | A | V | V | V | V | V | V |
| W2-31 | Wheat | / | / | / | / | / | / | / | / | / | / | / | / | / | / | / | / | / | / | / | / | / | / | / | / |
| W2-32 | Wheat | V | A | A | A | V | A | A | V | A | A | V | V | A | A | V | V | A | A | V | A | V | V | V | A |
| W2-33 | Wheat | V | A | A | A | V | V | V | V | A | A | A | A | A | V | V | V | A | V | V | V | V | V | V | A |
| W2-34 | Wheat | V | A | A | A | A | V | A | A | A | A | V | V | A | A | V | V | A | A | A | A | V | V | V | A |
| W2-35 | Wheat | V | V | V | A | A | V | A | V | A | A | V | V | A | A | V | A | A | A | V | V | V | V | A | A |
| W2-36 | Wheat | V | V | V | A | V | V | A | V | A | A | V | V | A | V | V | V | V | V | V | V | V | V | V | A |
| W2-37 | Wheat | V | A | A | A | V | V | A | V | A | A | V | A | A | A | V | V | A | V | V | V | V | V | V | A |
| W2-38 | Wheat | V | A | A | A | A | V | A | V | A | A | A | A | A | A | V | A | V | V | V | V | V | V | V | A |
| W2-39 | Wheat | V | V | V | A | V | V | A | A | A | A | V | A | A | A | V | A | A | A | V | V | V | V | V | A |
| W2-40 | Wheat | V | V | V | A | V | A | A | V | V | A | V | A | V | V | V | V | A | V | V | V | V | V | A | V |
| W2-41 | Wheat | V | V | V | A | V | V | A | V | V | A | V | A | V | V | V | V | A | V | V | V | V | V | V | V |
| W2-42 | Wheat | V | V | A | A | V | V | A | V | V | A | V | V | V | V | V | V | A | V | V | V | V | V | V | V |
| W2-43 | Wheat | V | A | V | A | V | V | A | V | V | A | A | A | V | V | V | V | A | V | V | V | V | V | V | V |
| W2-44 | Wheat | V | V | V | A | V | V | A | V | V | A | A | A | V | V | V | V | A | V | V | V | V | V | V | V |

^a^ Isolates of B1-1 to B1-16 of *Puccinia. striiformis* f. sp. *tritici* recovered from rusted barberry on 11 June, 2018, and those of B2-1 to B2-13 from barberry on 25 June, 2018; Isolates of W1-1 to W1-10 of *Puccinia. striiformis* f. sp. *tritici* collected from wheat in the vicinity of barberry bushes on 17 June, 2018, and those of W2-1 to W2-44 from nearby wheat on 25 June, 2018.

^b “/”^ phenotype data missing.
